# Optimization of moderators and beam extraction at the ESS^1^

**DOI:** 10.1107/S1600576718002406

**Published:** 2018-03-12

**Authors:** Ken Holst Andersen, Mads Bertelsen, Luca Zanini, Esben Bryndt Klinkby, Troels Schönfeldt, Phillip Martin Bentley, Jan Saroun

**Affiliations:** a European Spallation Source ERIC, PO Box 176, Lund 221 00, Sweden; b Niels Bohr Institute, University of Copenhagen, Copenhagen, Denmark; c Technical University of Denmark, Lyngby, Denmark; d Nuclear Physics Institute, Rez, Czech Republic

**Keywords:** water moderators, *para*-hydrogen moderators, low-dimensional moderators, pancake moderators, butterfly moderators, brilliance transfer, neutron instruments

## Abstract

A global approach coupling the moderator to the beam extraction system has been applied for the design optimization of the thermal and cold moderators of the European Spallation Source (ESS), which will be the brightest neutron source in the world for condensed-matter studies. The design is based on the recently developed high-brightness low-dimensional moderator concepts. *Para*-hydrogen is used for the cold neutron source, while thermal neutrons are provided by moderation in water. The overall moderation configuration was chosen in order to satisfy a range of requirements on bispectral extraction, beamport configuration and instrument performance. All instruments are served by a single moderator assembly above the target, arranged in a ‘butterfly’ geometry with a height of 3 cm. This was determined to be the optimal height for trade-off between high brightness and efficient guide illumination, by analysis of the performance of 23 instruments, based on the reference suite of the ESS Technical Design Report. The concept of ‘brilliance transfer’ is introduced to quantify the performance of the neutron optical system from the source to the sample. The target monolith incorporates a grid of 42 neutron beamports with an average separation of 6°, allowing a free choice between cold and thermal neutron sources at all instrument positions. With the large number of beamports and the space below the target available for future moderators, ample opportunities are available for future upgrades.

## Abstract 2

All instruments at the European Spallation Source (ESS), Lund, Sweden, are served by a carefully optimized moderator assembly, providing world-leading performance and excellent flexibility and upgradeability.

## 1. Introduction   

The European Spallation Source (ESS) is currently under construction in Lund, Sweden, and is expected to come on-line in the early 2020s, starting its user program in 2023. As a next-generation high-brightness neutron facility, it aims for world-leading performance for all its instruments. Central to this ambition is the brightness of the neutron source itself, coupled with an efficient and flexible neutron beam extraction and transport system to deliver the highest possible flux to the instruments.

**Figure 1:**
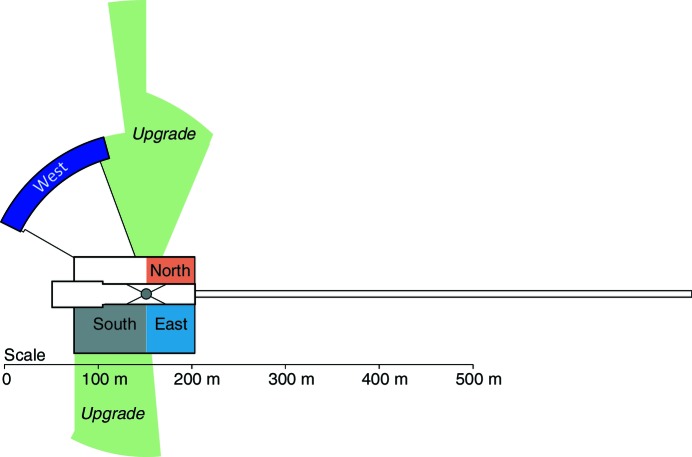
Schematic layout of the instrument halls, divided into instrument sectors labelled by their approximate compass directions relative to the target station, which is shown as a grey circle. The proton accelerator is indicated by the long horizontal rectangular building on the right. Potential upgrade areas for instruments outside the current instrument halls are indicated in green.

The technical and scientific scope of the ESS were described in the ESS Technical Design Report (TDR) (Peggs, 2013 ▸). It will be a 5 MW long-pulse neutron facility serving 22 instruments with a repetition rate of 14 Hz. The neutrons are produced by spallation from 2 GeV protons impinging on a rotating tungsten target using a pulsed proton beam with a proton pulse length of 1/(25 × 14 Hz) = 2.857 ms, where 1/25 is the duty cycle – the ratio between the pulse length and the repetition period. A number of experimental halls house the instruments, as shown in Fig. 1 ▸, together with a number of upgrade areas which are kept clear for future instrument hall expansions.

As is done at present-day accelerator-based neutron sources, the fast neutrons produced in the target are slowed down in moderators embedded in a reflector around the target to energies appropriate for the science to be performed on the instruments. The shielding around the target is penetrated by a number of beamports which allow extraction of the thermal­ized neutrons from the moderators towards the instruments.

At today’s pulsed neutron sources, the spectral and resolution needs of each instrument are satisfied by providing a number of moderators at different temperatures and with different types of neutron absorber (known as decoupling and poisoning) to tailor the neutron pulse width. At those facilities, the choice of beamport for an instrument determines which moderator and hence which spectral distribution and pulse widths are available to the instrument.

Whereas most existing spallation neutron sources use a proton pulse typically less than a microsecond in length, much less than the slowing-down time of the neutrons in the moderators, the ESS will be a long-pulse neutron source, with a pulse length greatly exceeding the moderation time. As a consequence, the primary means of selecting the neutron pulse width is to use a pulse-shaping chopper close to the neutron source, allowing the instrument to adjust its resolution in a much more flexible manner than at short-pulse sources. This also avoids the need for installing an array of decoupled and poisoned moderators providing different pulse widths, which reduces the peak brightness to increase resolution, as is done on current short-pulse sources. At the ESS, all beamports can provide high or low resolution by choosing to use a pulse-shaping chopper when required, while still benefitting from the high peak brightness of a coupled moderator.

An additional degree of flexibility is provided at the ESS by installing cold and thermal sources next to each other, both of which are viewable from all beamports. This allows all instruments to access the spectrum they need by tilting their neutron optics to point at the desired source and, if required, employing a bispectral switch system (Mezei & Russina, 2003 ▸) to stitch the cold and thermal spectra together and increase the available bandwidth. This became a design requirement on the moderator and beam extraction system from a very early stage in the project – to allow for each beamport a free choice of neutron spectrum, whether cold, thermal or bispectral – thus maximizing the flexibility available to instrument designers and hence the instrument performance.

Since the lifetime of the ESS is expected to exceed significantly the lifetime of any individual instrument, this high degree of flexibility in choosing the spectral and resolution characteristics at each beamport will allow for optimal design of all instruments, including future, as yet unknown, instruments, rather than hardwiring the ‘day one’ instrument suite into the layout of the facility.

The target monolith is designed with more beamports than the 22 needed for the initial scope, allowing for potential upgrades to the facility in the areas marked in Fig. 1 ▸.

With an average angular separation of 6° between adjacent beamports distributed over two 120° wide openings in the reflector, there will be 42 beamports at the ESS. Instruments in the West and South sectors are eventually expected to populate all beamports, while not all beamports in the North and East sectors will be usable for instruments at the same time, owing to the shorter length in these sectors which will in some cases cause the lateral size of instrument components (*e.g.* shielding) to block the view of neighbouring beamports.

The instrument suite of the ESS had not been finalized at the time of writing the TDR, which instead assembled a reference suite of 22 instruments covering the main science cases for the ESS and aimed to satisfy the diverse range of user communities of neutron beams. At the time of writing this article, decisions on the first 15 of these 22 instruments have been made, confirming that the TDR predictions were rather good – all 15 can be found in the reference suite.

The facility was designed around the TDR reference suite, in terms of both instrument performance and auxiliary facilities, such as laboratories, while retaining good flexibility for additional, as yet unknown, instruments and expansion possibilities.

The TDR design of the moderators employed volume *para*-hydrogen moderators, as pioneered and implemented at J-PARC (Kai *et al.*, 2004 ▸), with room-temperature water wings acting as the sources of thermal neutrons. The obtained time-averaged cold neutron brightness was very close to that of the ILL (Institut Laue–Langevin, 2008 ▸).

Shortly after publication of the ESS TDR, it was realized (Batkov *et al.*, 2013 ▸) that significant increases in both cold and thermal neutron brightness could be achieved by reducing the height of the moderators. This prompted a design research effort that led to several options for low-dimensional moderators for the ESS (Zanini *et al.*, 2014 ▸, 2015 ▸, 2016 ▸, 2018 ▸). The brightness of the source is a fundamental ingredient, but the important quantity for the instruments is the flux on the sample within the required phase space. The design of the optimal moderator must follow a holistic approach, by studying for each moderator configuration the resulting neutron flux at the sample. The configuration of the beam extraction system and the coupling with the optics must be taken into account. By following this approach, one can determine the optimal moderator vertical and horizontal dimensions, which dictate the moderator configuration, eventually leading to a choice among the different options. The route to the optimal source configuration must therefore go through several iterations where the configurations of the moderators, beam extraction system and optics mutually influence each other. The present paper describes how this approach, implemented for the specific case of the ESS, has led to an optimized moderator and beam extraction system.

The size of the neutron source, the size of the neutron guide that it feeds, the distance between them, and the grazing angle and reflectivity profile of the mirror surface of the guide together determine the efficiency of the beam extraction system. The beam extraction and transport system must be designed to maximize the overall transport efficiency of neutrons within the required phase space at the sample, whilst minimizing the number of neutrons outside this phase space (which increase the experimental background), and at the same time matching the phase-space dimensions and homogeneity to experimental requirements.

The beam extraction efficiency suffers when the source is reduced to a size similar to or smaller than the opening of the neutron guide. This results in a trade-off when reducing the source size, between the resultant brightness increase and the loss of beam extraction efficiency. This paper considers those trade-offs for a full suite of instruments, based on the TDR reference suite. It can be expected that the optimum trade-off will be different for each instrument, as each has different requirements on beam divergence, beam size and wavelength spectrum at the sample.

Various moderator geometries were considered for the ESS, starting with volume *para*-hydrogen and then flattened ‘pancake’ moderators, which are discussed in §2 of the present paper, as a means of benefitting from the increased brightness when reducing the moderator height. §3 describes the instrument suite used for analysing the performance and optimizing the design of the moderators. The concept of ‘brilliance transfer’ is introduced in §4 to quantify the loss of beam extraction efficiency when reducing the moderator size. §5 provides a quantitative analysis of the trade-off between source brightness and brilliance transfer for each instrument, resulting in the choice of an optimum moderator height of 3 cm. The issue of beam extraction is considered for the pancake moderator geometry in §6, while in §7 the ‘butterfly’ moderator geometry is introduced as a solution to the beam extraction problem. §8 concludes the paper with a short summary and the outlook for further upgrades.

## 2. From volume to low-dimensional moderators   

The performance of a neutron source is determined by its brightness and its geometry. For neutron sources, the terms ‘brightness’ and ‘brilliance’ are used interchangeably and are usually given in units of n cm^−2^ s^−1^ sr^−1^, or n cm^−2^ s^−1^ sr^−1^ Å^−1^ for the spectral brightness (brightness per unit wavelength). At a pulsed source, a distinction is made between the peak brightness, *i.e.* the maximum instantaneous brightness during a pulse, and the time-averaged brightness, for which the instantaneous brightness is averaged over a full repetition period. They are given in the same units. For a long-pulse source such as the ESS, the ratio of the time-averaged to the peak brightness is the duty cycle (the ratio between pulse length and repetition period) which is equal to 1/25 for the ESS.

**Figure 2:**
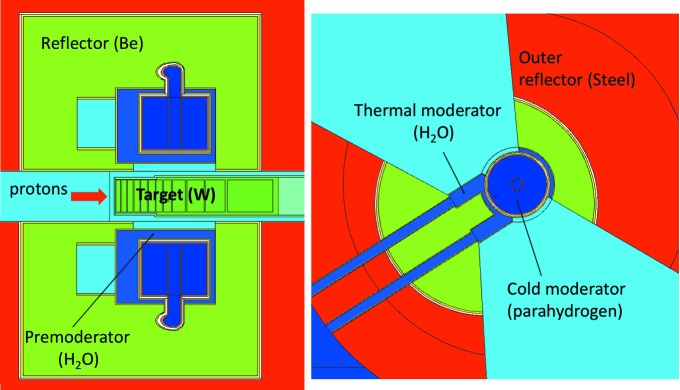
The TDR moderator geometry, from Zanini *et al.* (2014 ▸). (Left) Vertical cut. (Right) Horizontal cut through the upper moderator. The cold moderator has a diameter of 16 cm and the water wings are 11 cm wide. Both are viewable over a height of 12 cm. The lower moderator assembly is identical but is rotated clockwise by 60°, so that, between the two moderator assemblies, they allow instruments in 120° wide segments on both sides of the proton beam axis.

The TDR moderator (Magán *et al.*, 2013 ▸) design consists of volume *para*-hydrogen moderators above and below the target, each viewable in two 60° sectors, as indicated in Fig. 2 ▸.

The TDR moderator design incorporated slab-shaped water moderators at the side of each hydrogen moderator, so as to allow a view of both a cold and a thermal source for each beamport. The principle of a volume *para*-hydrogen moderator is that it provides a large moderating volume for the neutrons being slowed down by multiple collisions with the H_2_ molecules. The advantage of *para*-hydrogen over *ortho*-hydrogen is that, once the neutron energy falls below about 50 meV (Grammer *et al.*, 2015 ▸), the very large scattering cross section of the H_2_ molecules suddenly drops by more than an order of magnitude, making the moderator almost transparent and allowing the moderated neutrons to escape towards the neutron instruments. In the case of *ortho*-hydrogen, thermal­ization to cold energies occurs *via* recoil scattering with the free-gas hydrogen atoms. However, this cross section increases with decreasing neutron energy, *i.e.* there is not the drop observed for *para*-hydrogen. The implication of this behaviour is that neutrons will have many more collisions before exiting the moderator, with higher chances of being absorbed.

Alternative designs to the TDR were considered, following the finding (Batkov *et al.*, 2013 ▸) that significant gains in cold neutron brightness could be made by reducing the height of the *para*-hydrogen volume.

**Figure 3:**
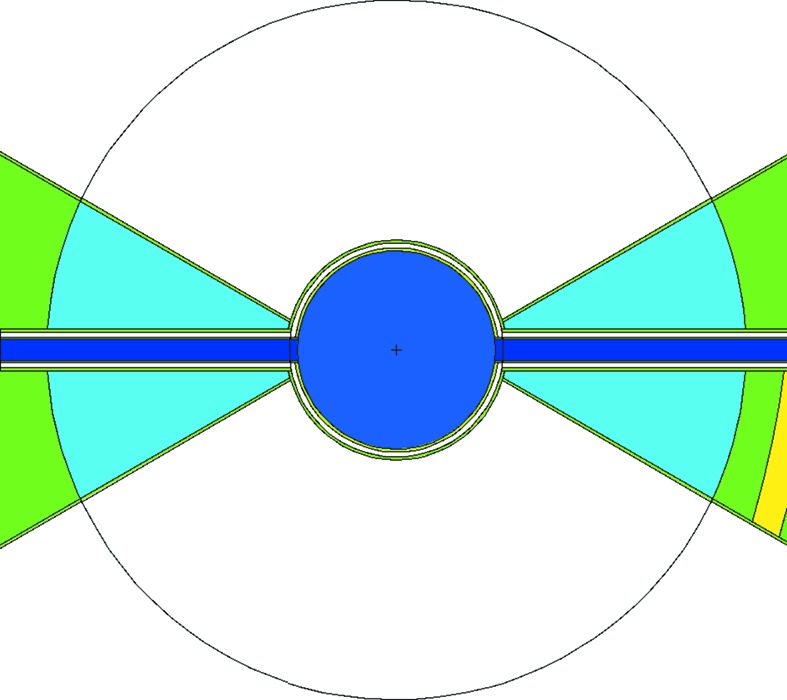
Horizontal slice through the pancake moderator geometry. The *para*-hydrogen moderator is shown as a dark-blue disc, while the four light-blue triangles represent the water wings. The green segments show the steel structure around the moderator. Not shown in the figure are the target (below the moderator) and the beryllium reflector (above). The proton beam is incident on the target from the right.

**Figure 4:**
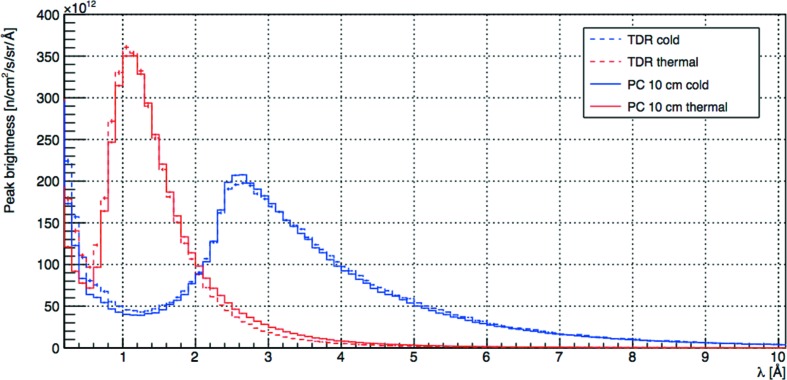
Peak brightness at 5 MW of the TDR and 10 cm pancake thermal and cold moderators. All curves correspond to a beamport in the centre of the reflector opening and are averaged over the full viewable height and a width of 6 cm.

The first envisaged implementation of this performance gain was the ‘pancake’ moderator geometry (Zanini *et al.*, 2014 ▸), outlined in Fig. 3 ▸. The pancake moderator assembly consists of a cylindrical *para*-hydrogen moderator with a diameter of 20 cm – somewhat wider than the TDR version. This is flanked on both sides by large triangular cross section water moderators, extending to 25 cm from the edge of the cold moderator. It should be noted that the effective width of the water moderators is significantly less than their physical size, as their brightness varies strongly in the horizontal direction, peaking at the junction with the cold moderator and falling away by 50% within about 12 cm. The pancake moderator assembly can be viewed *via* two symmetrically arranged 120°-wide openings in the reflector. The 10 cm high version of the pancake moderator provides almost identical brightness to the TDR moderator, as shown in Fig. 4 ▸.

**Figure 5:**
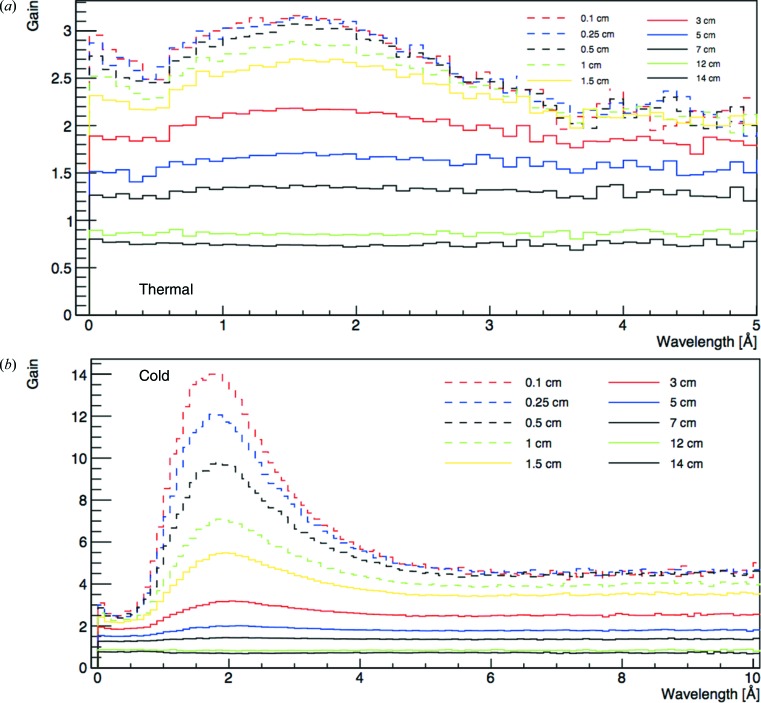
Gain factors for (*a*) the thermal and (*b*) the cold moderators (pancake geometry) as a function of wavelength, relative to the 10 cm high pancake moderator, after Zanini *et al.* (2018 ▸). As for Fig. 4 ▸, the gain factors correspond to a beamport in the centre of the reflector opening and are obtained by averaging over the full moderator height and a width of 6 cm.

The increase in neutron brightness as the height of the pancake moderator is reduced is shown in Fig. 5 ▸, in terms of a gain factor relative to the 10 cm high version, for a series of reduced heights with the same geometry otherwise. From Fig. 5 ▸ we see that the gain for the cold pancake moderators compared with the TDR geometry is about a factor of three for a 3 cm high moderator, highest near the peak of the cold spectrum and slightly lower for longer and shorter wavelengths. The thermal gain factors are somewhat lower: about two for the same moderator height, with a weaker wavelength dependence. The gain arises from a combination of two effects. The first is an edge-enhancement effect which applies mainly in the case of *para*-hydrogen: the surface brightness is always higher near the boundaries with the pre-moderator or reflector immediately above and below the moderator. This is where most of the under-moderated neutrons arrive, or where they are reflected back in after partial moderation within the hydrogen volume. Neutrons in the 50–100 meV range will have a mean free path of about 1 cm in hydrogen. When, after additional collisions, they reach the cold energy range, the mean free path abruptly increases to the order of 10 cm, due to the abrupt decrease in the inelastic cross section. They will then leave the moderator and contribute to the increase in the edge surface brightness. Reducing the moderator height thus increases the relative proportion of brighter edges. Increasing the moderator diameter from the 16 cm of the TDR further enhances this effect. Since, for pure *para*-hydrogen, the moderator is quasi-transparent for neutrons with energies below the *ortho*–*para* transition of 14.7 meV (the main remaining reaction being capture in hydrogen nuclei with the typical 1/*v* cross section), neutrons which are moderated to below this threshold can freely make it to the viewable surface, starting from almost any depth inside the volume, increasing the cold neutron brightness as the diameter is increased. Neutron absorption does, however, result in a decrease in brightness above a diameter of about 24 cm; the maximum brightness is achieved for a diameter between 20 and 24 cm (Zanini *et al.*, 2014 ▸)

The second effect is a reduction in leakage and applies to both the cold and thermal moderators. Partially moderated neutrons will leave the moderator through all surfaces. The pre-moderator and reflector around, above and below the moderator are designed to maximize the probability of reflecting those neutrons back in to continue the moderation process. However, partially moderated neutrons escaping out through the viewed surface are unlikely to be reflected back in and are lost to the system. By reducing the height of the moderator, the sizes of the openings in the reflector are also significantly reduced, reducing the leakage of under-moderated neutrons and increasing the likelihood that they are reflected back in if they do leak out. This reduction in leakage is so large that the horizontal opening of the pancake moderator can be increased to the full 2 × 120° needed to allow a single moderator assembly to serve all instruments, eliminating the need for moderators both above and below the target as in the TDR.

## 3. Instrument requirements   

The impact on the instrument performance of the combined increase in moderator brightness and change in moderator geometry, outlined in §2, depends strongly on the requirements of each instrument in terms of divergence, beam area and wavelength band. These are summarized in Table 1 ▸ for the 23 instruments covered in this study.

Most of the instruments in Table 1 ▸ can be found in the ESS Technical Design Report (TDR), to which the reader is referred for more information on the individual instruments. The changes were imposed by the availability of instrument teams to provide the initial information for the present study and to evaluate the results, and by changes to instrument designs since publication of the TDR. On balance, the instrument list in Table 1 ▸ is likely to be closer to the instrument suite which will eventually be built, as it is more recent and has taken some of the subsequent instrument decisions into account.

The first column of the table gives the instrument name, together with a brief description, matching the instrument name given in the TDR. Instruments indicated by an asterisk (*) in the table are substantially different from the TDR instruments, as described in the table caption. The second column gives the length of the instrument from the moderator to the sample, with a few exceptions which are stated. In the case of ODIN, the imaging beamline, it is the distance to the pin-hole defining the instrument resolution. For HOD, a crystal-monochromator diffractometer, it is the distance from the moderator to the monochromator. In the case of the two spin–echo instruments, the moderator–sample distance is given as the sum of the distance from the moderator to the end of the guide and the distance from the guide end to the sample, imposed by the spin–echo precession region.

Columns 3 and 4 of Table 1 ▸ give the required beam area and divergence of useful neutrons at the sample. This is the volume of phase space over which the flux needs to be maximized. In the case of ODIN, it is given at the pinhole rather than the sample position, while for HOD it is given at the monochromator position. The n-nbar instrument is treated differently from the others: it is a 300 m long instrument proposed to search for neutron–antineutron oscillations, similar to an instrument built and operated at the ILL in the early 1990s (Baldo-Ceolin *et al.*, 1994 ▸). It is designed to extract as many neutrons as possible into an extremely large beam, of the order of 1 m^2^ in cross section. Its figure of merit is not the flux at the sample integrated over a given phase-space volume, as for most of the other instruments, but the spectral source brightness integrated over the viewed surface and multiplied by the wavelength squared. More information can be found in the article by Milstead (2015 ▸).

The last column indicates whether the instrument uses cold, thermal or bispectral beam extraction and states the wavelength band of interest. The volumes of phase space and the wavelength bands were supplied by the instrument teams (ESS Design Update^2^). In most cases, they are rather conservative, corresponding to larger than average samples and/or a divergence towards the larger end of the range of divergences that are expected to be used. This will have the effect of slightly biasing the result towards larger moderators.

For bispectral instruments, there may be two different phase-space requirements, depending on whether the instrument operates differently when using cold and thermal neutrons.

## 4. Method of performance analysis   

The analysis generally seeks to maximize the flux at the sample, integrated over the area and divergence interval shown in Table 1 ▸. The flux at the sample is the product of the brilliance of the source, integrated over the appropriate area and divergence interval, and the transmission of the system, defined over that same phase space (beam area and divergence interval). We denote the transmission defined in this way as the ‘brilliance transfer’ (BT) of the system, and note that it is defined only for a particular beam area and divergence interval.

This separation of the problem into source brilliance and brilliance transfer allows a simple separation between, on the one hand, the brightness of the source and, on the other, the geometry and other neutron transport properties of everything happening between the source and the sample. The BT is a measure of how close the neutron transport system is to the upper limit given by Liouville’s theorem.

Liouville’s theorem (Liouville, 1838 ▸) states that, for a conservative system, the phase-space density along the trajectories of the system is constant. For beam transport, this means that the flux per unit phase space (area, divergence, wavelength, time), *i.e.* brightness, evaluated anywhere along the beam will be the same as at the source, if there are no losses. Any imperfections in the system, such as gaps (*e.g.* between the source and guide start) or guide reflectivities less than unity, can lead to a BT which is significantly less than one. It is a straightforward and dimensionless measure of beam transport all the way from the source to the sample.

**Figure 6:**
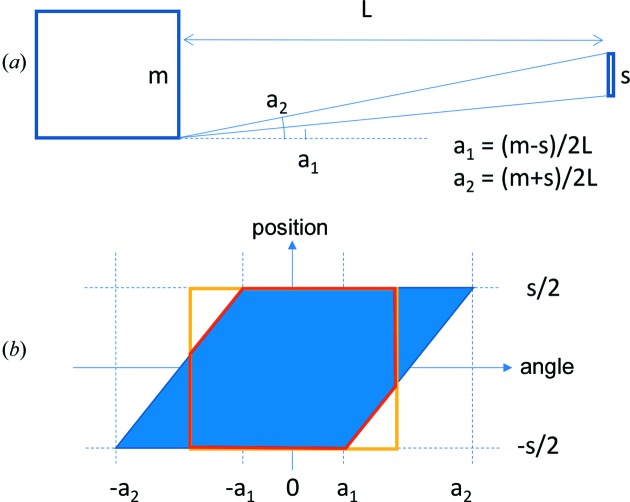
Method of evaluating brilliance transfer (BT) for a simple model two-dimensional system. (*a*) Sketch of the system, consisting of a moderator of height *m* on the left, illuminating a sample of height *s* at a distance *L*. (*b*) The corresponding acceptance diagram. The blue parallelogram shows the area of phase space incident on the sample. The yellow rectangle shows the area of phase space (beam height and divergence interval) for which we wish to evaluate the BT. The intersection of the two areas is outlined in red.

An illustration of the BT for a simple model system is given in Fig. 6 ▸, using the formalism of acceptance diagrams (ADs) (Carpenter & Mildner, 1982 ▸; Bentley & Andersen, 2009 ▸; Bertelsen & Lefmann, 2016 ▸). The BT of the very simple geometry shown in Fig. 6 ▸ can be calculated straightforwardly, as indicated in the figure. It is the ratio of the area of the red polygon to that of the yellow rectangle. In this example, it can be seen to be about 90%. This is thus an example of a configuration which is close to fully illuminated; the moderator is considerably taller than the sample, and their separation is small enough that the illumination angles shown in the figure are comparable to the acceptance angle for defining the BT. As a consequence, the difference in the areas of the red and yellow polygons is small, indicating that most of the sample is illuminated by most of the divergence which can be accepted. If the moderator height is reduced, the sample height increased or their distance increased, the illumination will get worse, resulting in a lower BT, as illustrated in the following example.

**Figure 7:**
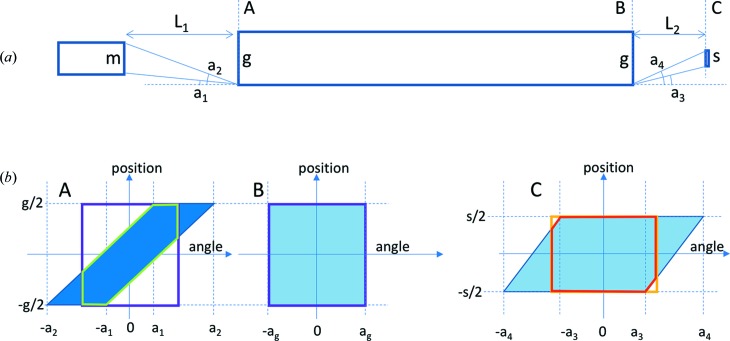
Method of evaluating the BT for a more realistic two-dimensional system. (*a*) Sketch of the system, consisting of a moderator of height *m* illuminating a guide of height *g* starting at a distance *L*_1_ from the moderator, where *g* is greater than *m*. A sample of height *s* is placed at a distance *L*_2_ from the end of the guide. The guide is assumed to be long compared with the gaps *L*_1_ and *L*_2_. (*b*) Acceptance diagrams for the positions labelled A, B and C in panel (*a*), corresponding to the guide entrance, guide exit and sample position, respectively. The blue areas show the areas of phase space at those positions. The purple rectangle shows the phase-space acceptance of the guide, and the intersection of the blue and purple areas is outlined in green. The yellow rectangle shows the area of phase space for which we wish to evaluate the BT. The intersection of the blue and yellow areas is outlined in red.

A more complicated two-dimensional system is shown in Fig. 7 ▸. It consists of a moderator, a neutron guide and a sample, separated by gaps. In this example, the guide entrance is under-illuminated, resulting in a rather strong correlation between position and divergence.

The BT of the system shown in Fig. 7 ▸ takes a little bit more effort to illustrate than the preceding example. The left-hand acceptance diagram of the figure shows the phase space incident on the guide entrance as a blue parallelogram. Since the guide entrance in this example is larger than the moderator, the guide is rather under-illuminated. This can be seen from the shape of the blue parallelogram, which only fills about half of the purple rectangle indicating the phase-space area which can be accepted by the guide. The guide acceptance is bounded in space by its height and in divergence by the critical angle of total reflection of the guide coating, denoted *a*_g_ in this example. Note that the guide height and critical angle are roughly matched in this setup: increasing the guide height or critical angle independently would not significantly increase the area of phase space accepted at the entrance.

During transport through the guide, the neutrons will reflect many times from both the top and bottom of the guide, resulting in mixing and a decorrelation between position and trajectory angle. The neutrons accepted into the guide, indicated by the green polygon at A, become sheared and the phase-space regions bounded by the mirror surfaces are reflected and attenuated. The end result is that the large white regions of unfilled phase space within the purple rectangle are stretched thinly and distributed throughout the guide acceptance area as diagonal stripes, and a coarse resolution measurement reveals a smooth phase space of reduced spectral weight, as shown at B. This results in a dilution of the phase-space density, corresponding to the ratio of the green and purple polygons at A, as illustrated by the lighter blue colour filling the rectangle at B.

Finally, the illumination of the sample is shown by the acceptance diagram at C. The BT can then be evaluated by calculating the area of the red polygon, indicating the overlap between the phase space delivered to the sample and the phase space (beam height and divergence) for which the BT is to be evaluated, shown as the yellow rectangle, and dividing by the area of the yellow rectangle. This area then needs to be scaled down to account for the dilution of phase-space density resulting from the under-illumination of the guide entrance and the subsequent mixing in the guide. That corresponds to multiplying by the ratio between the green and purple areas. By simple visual inspection it appears to be about 50%.

In both the above examples, the BT is shown for a simple straight two-dimensional (vertical) system only. Most three-dimensional guide systems can be evaluated straightforwardly by treating them as two independent two-dimensional systems, one horizontal and one vertical. The BT of the three-dimensional system is then the product of the two two-dimensional systems.

Clearly, more sophisticated methods of calculating the transport efficiency are required for complex geometries. In practice, the brilliance transfer can be evaluated straightforwardly using a three-dimensional ray-tracing programme such as *McStas* (Lefmann & Nielsen, 1999 ▸; Willendrup *et al.*, 2014 ▸), *Vitess* (Wechsler *et al.*, 2000 ▸), *Restrax* (Šaroun & Kulda, 2008 ▸) or *NADS* (Bentley & Andersen, 2009 ▸). Using an arbitrary source brightness, the flux at the sample is evaluated and then integrated over the appropriate beam area and divergence interval. The BT is then given by the ratio of this integral to the same integral performed over the same phase space on the moderator face.

The spectral neutron current (*i.e.* area-integrated flux) at the sample can be expressed as
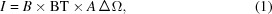
where *B* is the spectral brilliance of the source, usually given in units of n cm^−2^ s^−1^ sr^−1^ Å^−1^, BT is the dimensionless brilliance transfer from the source to the sample, *A* is the desired beam area at the sample and 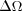 is the solid angle, *i.e.* the two-dimensional divergence range, to be delivered to the sample. The BT is evaluated for the phase space given by *A* and 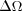.

When the flux at the sample is evaluated using one of the ray-tracing programmes, the BT can also be extracted using equation (1) and averaging the source brightness *B* over the full viewed area of the source.

For a straightforward comparison between instruments in this study, we define a dimensionless figure of merit (FoM) for each instrument, proportional to the flux at the sample, as follows:
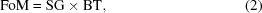
where SG is the source gain, *i.e.* the brilliance of the source relative to the brilliance of the TDR moderator. It is evaluated by integrating the source brightness over the relevant wavelength range of the instrument, shown in Table 1 ▸, and dividing by the same integral evaluated for the TDR moderator. The BT in this case should also be averaged over the relevant wavelength range.

An additional constraint is imposed by some instruments on the uniformity of the divergence distribution incident on the sample. As the guide becomes increasingly under-illuminated for small moderator heights, the gaps in phase space [the differences between the green and purple areas in Fig. 7 ▸(*b*)] at the guide entrance increase in size. This can end up resulting in visible gaps in phase space (divergence or spatial uniformity) at the sample position, if the guide is not sufficiently long to mix the incident phase-space gaps smoothly into the phase-space volume of interest at the sample.

## 5. Determining the optimum moderator height   

The following analysis will evaluate the FoM for the pancake moderators as a function of moderator height. The source brightness for each moderator height is evaluated by multiplying the brightness curve for the 10 cm pancake moderator shown in Fig. 4 ▸ by the appropriate gain factors in Fig. 5 ▸. Gain factors were interpolated for intermediate moderator heights. The SG is then obtained by integrating that source brightness over the appropriate wavelength range and dividing it by the same integral evaluated for the 10 cm pancake moderator. For simplicity of comparison, the BT numbers which are also evaluated as a function of moderator height will be normalized with respect to the value obtained for the 10 cm pancake moderator. The FoM is thus constrained to be one for the 10 cm pancake moderator, by definition. We recall that the source brightness of the TDR and 10 cm pancake moderators are essentially the same, as shown in Fig. 4 ▸.

**Figure 8:**
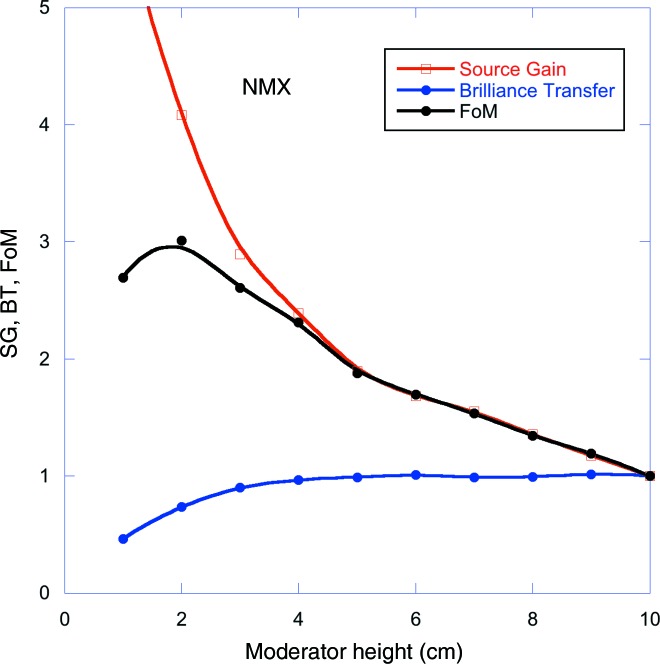
FoM, SG and BT for the NMX macromolecular crystallography instrument, for a guide system starting at 2 m from the moderator. Both the SG and BT have been normalized with respect to their value for the 10 cm moderator height.

An evaluation of the FoM, SG and BT for the NMX macromolecular crystallography instrument is given below as an example of the method. Starting with the 10 cm high pancake moderator, the guide system is optimized so as to maximize the flux at the sample within the desired phase-space volume given in Table 1 ▸, noting the resultant BT. The moderator height is then reduced and the guide geometry is re­optimized to maximize the BT to the sample again. The process is repeated for a range of moderator heights. The results are shown in Fig. 8 ▸.

As can be seen in Fig. 8 ▸, the SG increases monotonically as the moderator height is decreased, describing the increasing source brightness. The BT is independent of moderator height in the limit of large height. However, as the height is decreased below about 4 cm, the BT starts to decrease, largely because of under-illumination of the guide entrance. The FoM, which is the product of the two, thus displays a maximum at a moderator height of about 2 cm, providing a FoM of about three relative to the TDR moderator.

This analysis was performed on all the instruments listed in Table 1 ▸, representing a very large effort in terms of guide optimization, in order to find the guide providing the highest BT for each moderator height and each instrument. The scale of this work was made possible, in part, by a new development in instrument simulation, the *guide_bot* (Bertelsen, 2017 ▸) extension to *McStas*, which provides a framework for the automated optimization of guides within well defined and controllable boundary conditions.

For cold and thermal instruments, the FoM is calculated over an average of the wavelength range given in Table 1 ▸. For the bispectral instruments, the FoM was evaluated separately for the cold and thermal ranges and the results were then averaged to provide a single FoM for each moderator height.

In many cases, additional constraints on the optimization were placed on the guide system, other than those stated in Table 1 ▸. Such constraints include gaps in the guide system for accommodating choppers, guide size restrictions at those gaps in order to constrain the size of the choppers, guide curvature or kinking to eliminate short-wavelength neutrons, ending the guide a sufficient distance before the sample to allow for collimation or a bulky sample environment, and restricting the maximum guide width, height or supermirror coating in order to contain the cost. These and other constraints can be straightforwardly handled within *guide_bot*.

**Figure 9:**
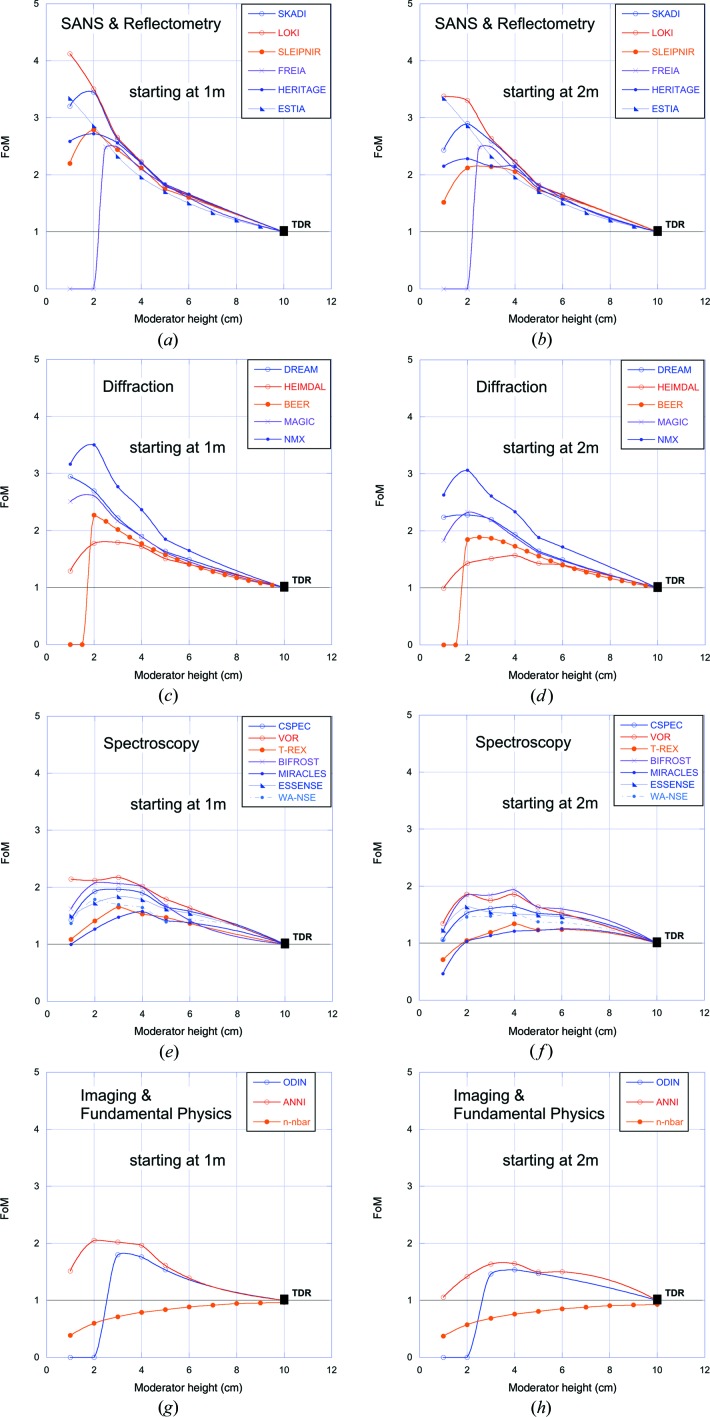
FoM as a function of moderator height for all the instruments in Table 1 ▸. (*a*), (*c*), (*e*), (*g*) With guides allowed to start at 1 m from the moderator. (*b*), (*d*), (*f*), (*h*) With guides constrained to start after 2 m from the moderator.

The resulting FoM curves for all the instruments are shown in Fig. 9 ▸. Analysis of the data shown in Fig. 9 ▸ reveals that the FoM of most instruments peaks for a moderator height between 2 and 4 cm, with a gain in performance relative to the TDR of typically a factor of 1.5–3. For a few instruments, the FoM can be seen to drop to zero below a critical moderator height. In these cases, the instrument teams judged that the resultant vertical divergence profile at the sample was un­acceptably non-uniform for the smallest moderator heights. Though the usual FoM was typically above one in these cases, it was manually adjusted down to zero to reflect the necessity of maintaining acceptable divergence uniformity. This is a fairly crude and conservative approach, as there is a significant amount of freedom available to shape the vertical divergence distribution by further adjusting the shape of the guide near the sample. That was felt to be beyond the scope of this study.

**Figure 10:**
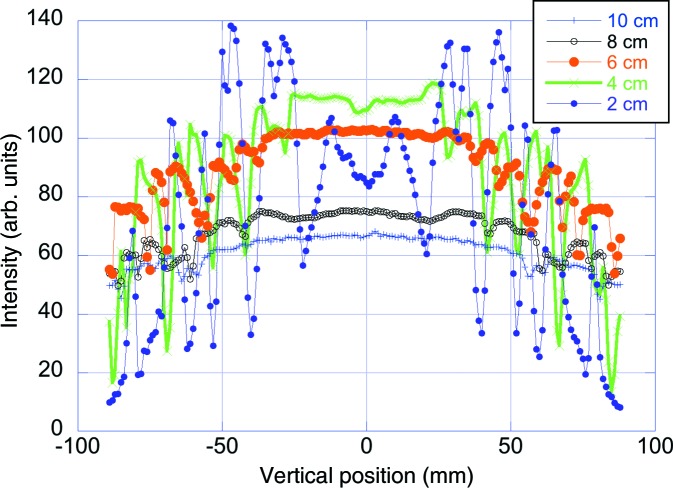
Horizontally integrated beam images at the detector position of ODIN, 10 m downstream of a 3 × 3 cm pinhole, for 2, 4, 6, 8 and 10 cm tall moderators.

As an illustration of this effect, the field of view on the ODIN instrument is shown in Fig. 10 ▸. The ODIN beam image illustrates how the intensity increases with decreasing moderator height, reaching a maximum at a moderator height of about 4 cm, while the beam profile becomes gradually less uniform as the moderator height is reduced, reflecting the increasing non-uniformity of the beam divergence. Rather than attempting a somewhat subjective incorporation of the divergence uniformity into the FOM, the instrument team made the judgement that the non-uniformity of the beam profile would result in unacceptably poor image quality for moderator heights of 2 cm and less. It was judged that, at larger moderator heights, the remaining non-uniformities could probably be dealt with by further adjustment of the guide profile. Continuing optimization work has later confirmed that good beam uniformity can be achieved for a moderator height of 3 cm.

All the guide optimizations were performed for two separate boundary conditions: with the instrument guide allowed to start at 1 m from the moderator and with the guide constrained to start after 2 m from the moderator.

It is seen in Fig. 9 ▸ that all instruments apart from n-nbar will benefit significantly in performance by viewing a 2–4 cm tall pancake moderator, compared with the TDR moderator. Most instruments will gain slightly in performance by being able to start their guide at 1 m from the moderator.

Small-angle neutron scattering (SANS) and reflectometry instruments stand to gain very little by moving the guide entrance closer to the moderators. This is not surprising, as they typically use a rather small volume of phase space, allowing the guide entrance to be fairly well illuminated even at a distance of 2 m. The situation is similar for the diffraction instruments, which typically improve by less than 10% when moving to 1 m. The spectroscopy instruments gain a little more, as they usually accept a larger phase-space volume in order to compensate for the losses in counting rate inherent in analysing the energy transfer. The biggest winners would be MIRACLES and T-REX, which both stand to gain 30–40% by moving their guide entrance from 2 m to 1 m from the moderators. The gains for the fundamental physics and imaging instruments are similar to those of the spectroscopy instruments.

**Figure 11:**
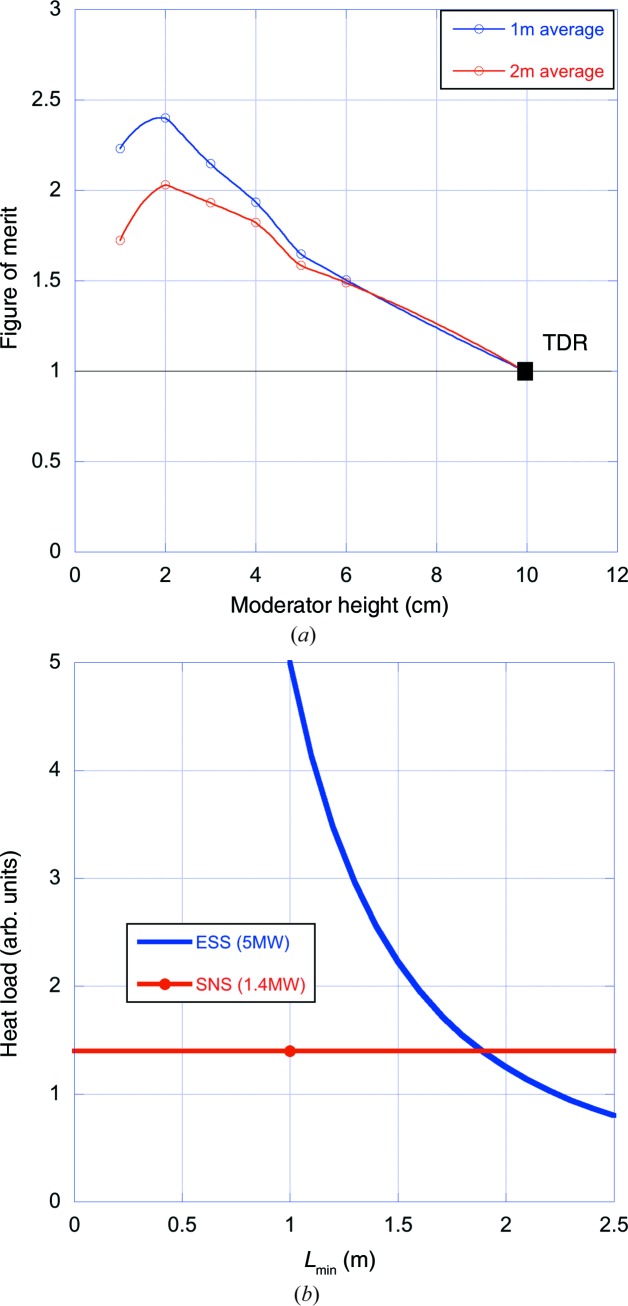
(*a*) FoM curves averaged over all instruments in Fig. 9 ▸ for minimum source–guide distances of 1 and 2 m. FoM values which were set to zero in Fig. 9 ▸, as described in the text, have been excluded from the averages. (*b*) The heat load at the guide entrance as a function of guide start, relative to the Spallation Neutron Source (SNS, Oak Ridge, Tennessee, USA) at 1.4 MW and assuming that it scales as *P*/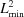 where *P* is the time-averaged power of the source and *L*_min_ is the minimum guide distance. *L*_min_ at the SNS is 1.0 m.

In order to give an overview, the gains averaged over all instruments are shown in Fig. 11 ▸(*a*). Here, it is seen firstly that the optimum moderator height does not change significantly by allowing the guides to start closer to the moderator. The optimum is at 2 cm for both guide distances. Secondly, it is clear that the average performance improvement from allowing a closer approach is of the order of 10–20% for 2–3 cm tall moderators, while the heat load and radiation damage at the front of the guides will increase by about a factor of four, as indicated in Fig. 11 ▸(*b*).

Not considered so far is the fact that adjacent guides are likely to start interfering with each other laterally as they approach the moderator beyond the 2 m position, given the beamline angular separation of about 6°. Even ignoring issues relating to heat load and radiation damage, it is unlikely that separate guides can start at much closer than about 1.5 m in order to avoid collision with neighbouring guides. A possible solution would be to install a common guide for several beamports, consisting of just top and bottom faces, with a shape representing a compromise between the optimal geometries of the various individual guides which it replaces. The net performance increase would therefore be significantly less than the 10–20% evaluated for the case of individually optimized guides starting at 1 m.

We conclude that the potential gain in starting the guides at less than 2 m is likely to be less than 10%, which is not judged to be sufficient to justify the increased risk of damage to the guide front end.

The maximum in the average FoM curves shown in Fig. 11 ▸(*a*) is at a moderator height of 2 cm. However, this average excludes ODIN and FREIA, which did not find a suitable guide geometry at this height. It is difficult to see how a solution could be found for FREIA, where the effects of gravity start to limit the accessible wavelength range for very small moderator heights, thus reducing the dynamic range of the instrument. This is particularly problematic for FREIA, which is an instrument optimized for kinetic measurements on free liquid samples which needs the full wavelength range for its instantaneous *Q* range. For most of the other instruments, reducing the moderator height from 3 to 2 cm would result in significantly less uniform divergence distributions at the sample. Though only ODIN, and to some extent BEER, indicated that this degradation in beam quality would be unacceptable, it was deemed prudent not to push to the smallest possible moderator height, particularly since the difference in average performance between the 3 and 2 cm moderator heights is only about 5%. Given also the time pressure to fix the moderator geometry and the remaining uncertainties in performance, it was felt to be important to leave a reasonable safety margin. The moderator height was thus set to 3 cm.

## 6. Beam extraction considerations   

The preceding section has described how the moderator height was optimized. The analysis assumed a pancake geometry, but is valid for any geometry displaying the same variation in source brightness as a function of moderator height. The pancake moderator layout, however, suffers from two problems.

The first problem is one that is shared with the TDR geometry: the thermal moderators are placed rather far away from the geometric centre, the most intense part of the neutron production volume. Their performance is therefore somewhat penalized compared with the cold moderator. This was felt to be a reasonable compromise, since the majority of ESS instruments use a cold spectrum. There are, however, a significant fraction of bispectral instruments, as well as a minority of thermal instruments (see Table 1 ▸), giving rise to an effort to find ways of increasing the thermal brightness.

This problem was initially addressed by the design of a separate moderator assembly, optimized for high thermal brightness, termed the ‘optimized thermal’ geometry. This was expected to complement a pancake moderator above the target, by being placed below the target, and consisted of a rather compact water moderator, placed as close as possible to the most intense part of the neutron production volume in the target, with a somewhat compromised cold moderator on the side (Zanini *et al.*, 2014 ▸). Such an exercise was very useful for assessing the maximum achievable thermal brightness in the ESS configuration. It was found that the thermal brightness in this configuration reaches about 75% of the maximum achievable for a stand-alone thermal moderator. The cold brightness would, however, be penalized and efforts were made to seek alternative cold geometries, such as the ‘tube’ (a small cross section with a large depth), with a highly directional brightness enhancement along the tube axis. In the end, it was felt that none of these geometries was able to serve instruments on a sufficient number of beamlines.

**Figure 12:**
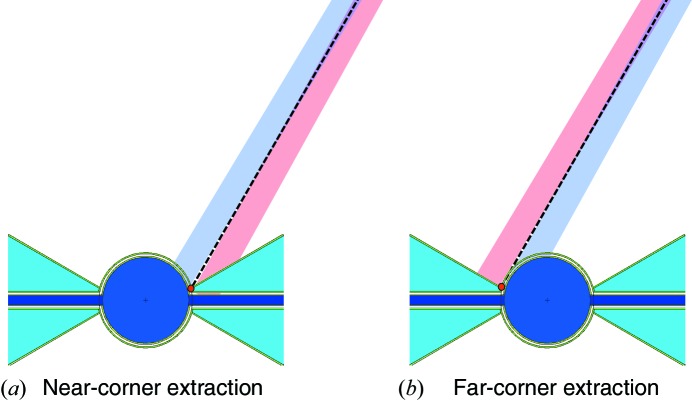
Beam extraction options for the pancake moderator, illustrated for a beamline in the middle of the North sector, *i.e.* at 60° counter-clockwise with respect to the incoming proton beam, incident from the right. The focal point for the North sector is shown as a red dot. (*a*) Near-corner extraction, such that each beamport axis points at the nearest cold–thermal junction. (*b*) Far-corner extraction, in which each beamport axis is oriented towards the more distant cold–thermal junction. In each case, the beamport axis is shown as a dashed black line, and the shaded light-blue and pink areas indicate the views of the cold and thermal sources, respectively, which can be achieved by tilting the instrument axis by 1° with respect to the beamport axis. The view areas shown are 7 cm wide.

The second problem is related to beam extraction and is inherent in the pancake geometry. In order to allow each beamport the option of both cold and thermal spectra, each neutron beamport should be oriented to point at the junction between the cold and thermal moderators, allowing a choice of either spectrum by tilting the guide slightly within the beamport insert to the left or right. This junction is termed the ‘focal point’ and serves as the origin of the beamport axis for all beamports within an instrument sector. We recall that the ESS has four instrument sectors, as shown in Fig. 1 ▸. A beamport in the upper 120° opening shown in Fig. 3 ▸ should thus be oriented towards either the cold–thermal junction on the left or that on the right of the hydrogen pancake. This is illustrated in Fig. 12 ▸.

We take the example of an instrument around the middle of the North sector, *i.e.* the instruments with beamport angles between 30 and 90° with respect to the proton beam, incident from the right in Figs. 3 ▸ and 12 ▸. If the beamport is pointing towards the nearest cold–thermal junction, as shown in Fig. 12 ▸(*a*), it will view the water wing at a glancing angle of the order of 30°, while its view on the cold moderator will be roughly perpendicular to the surface. The optimal orientation is always perpendicular. A perpendicular view of the cold moderator results in the greatest viewed depth of hydrogen, enhancing the pancake moderator effect. When a water wing is viewed at a glancing angle, it has the effect of reducing its projected width, exacerbating the problematic reduction in brightness with distance from the cold–thermal junction. This problem is most severe for the edge beamports, where the projected width of the water moderator falls to almost zero.

If that same beamport is now shifted to point to the cold–thermal junction on the other side of the pancake moderator, as shown in Fig. 12 ▸(*b*), the situation is reversed: it is now viewing the thermal wing perpendicularly, which is favourable. However, its view of the cold moderator is at a glancing angle, significantly reducing its brightness. In both cases, the shaded blue and pink areas in Fig. 12 ▸ indicate the source view which can typically be achieved by tilting and translating the neutron guide inside the beamport insert, so as to view the cold or thermal source, respectively. If far-corner extraction is chosen, the horizontal axis of the instrument would need to be rotated by more than 3° in order to achieve a perpendicular view of the cold moderator. Such a large rotation is not possible, given the constraints on the dimensions of the beamport inserts. The result is therefore a somewhat compromised brightness of the cold moderator, as shown further below.

The very low thermal brightness at the edge beamports for near-corner extraction essentially makes them unusable for thermal and bispectral instruments. Thus, the design requirement for the moderators of allowing cold, thermal or bispectral beams on all beamports is not satisfied. The far-corner extraction option is therefore the preferred beamport geometry for the pancake moderator, despite the roughly 10% loss of cold brightness for most beamports.

However, the far-corner extraction geometry has another difficulty: that of maintaining the large number of beamports envisaged. The 6° beamport separation at the ESS was chosen so as to maximize the number of usable beamports. In today’s pulsed spallation neutron sources, beamports tend to be separated by approximately 10–12°, owing to the lateral size of the instrument shielding. This accounts for the number of instruments (around 22) which can be found today at the target stations of ISIS, SNS and J-PARC. The short instrument sectors (North and East; see Fig. 1 ▸) at the ESS will house instruments of a similar length to those facilities. We therefore expect to use about one in two beamports in these two sectors, as the intermediate ones will be rendered unusable by the space taken up by the instrument shielding.

In the South and West sectors, however, the much greater instrument lengths at the ESS allow a smaller beamport separation. In the West sector, in particular, where the instruments are all approximately 165 m long, the size of the instrument hall can easily accommodate beamport separations down to even below 5°. The limitation here is set by the interference between the neutron choppers and other beamline components of adjacent instruments in the first metres near the target monolith. Studies were made which resulted in the judgement that the beamport separation at the ESS could be reduced, compared with other facilities, down to about 6° while still allowing installation and operation of instruments on adjacent beamports. In some cases, this results in the need to reduce the size of the chopper discs, shifting choppers radially in order to stagger them between adjacent beamlines, reducing the amount of standardization across chopper modules, and designing guides and downstream shielding so as to compensate for the increased cross-talk between beamlines resulting from the reduced amount of shielding between them near the monolith. These were felt to be acceptable compromises in order to keep a good upgrade path to increasing the number of instruments. As a final tweak, the beamport separations in the West and South sectors have been set to 5.7 and 6.3°, alternately, so as to reduce the overall amount of integration needed between adjacent beamlines; each instrument team can focus their integration efforts on the instrument on just one side.

The 6° beamport separation creates another difficulty: at the 2 m position (from target axis to guide entrance), the separation between adjacent beamports is only 21 cm. This compares with the typical width of a neutron guide (including substrate thickness) of the order of 8 cm. The remaining lateral space is fully taken up by the space needed for translating and/or rotating the guide axis, so as to view the cold or thermal source on either side of the beamport axis. The far-corner extraction geometry causes a clash of the beamports on either side of the perpendicular to the proton beam, as adjacent beamports switch from viewing one focal point to another. This was judged to result in the loss of 2–6 beamports overall (Bentley & Klinkby, 2015 ▸).

## 7. Butterfly moderators   

**Figure 13:**
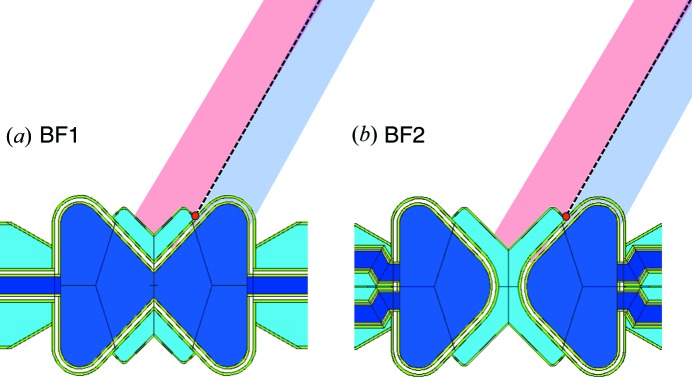
Horizontal slices through the butterfly geometries. (*a*) BF1 geometry. (*b*) BF2 geometry. The dark-blue volumes are *para*-hydrogen and the turquoise volumes are water. The proton beam is incident from the right. The focal point for the North sector is indicated with a red spot. The views of the cold and thermal sources are illustrated for the North beamport at 60° with respect to the proton beam, as for Fig. 12 ▸.

From the previous section, it appears that, for the pancake geometry, the number of beamports needs to be reduced, or else we accept a compromised thermal performance, particularly for instruments placed at beamports far from the perpendicular to the proton beam. The butterfly (BF) geometry (Schönfeldt, 2016 ▸; Zanini *et al.*, 2016 ▸, 2018 ▸) solves the beam extraction issues while also improving the brightness of the thermal moderator. It exists in two variants, BF1 and BF2, shown in Fig. 13 ▸.

The butterfly geometry is, at its origin, a variant of the pancake geometry: the *para*-hydrogen volume has been extended parallel to the proton beam and pinched in the middle, so as to allow for a V-shaped pair of water wings in the middle which are separate in the BF1 variant but join together in the middle in the BF2 variant, cutting the hydrogen volume in two. It improves the performance of the water moderator by placing the water wings in the central, most intense, part of the neutron production volume. The cold brightness is maintained for the beamlines at around the normal to the proton beam, by slightly increasing the hydrogen dimensions perpendicular to the proton beam. Beamlines around 45° see an enhanced cold brightness with respect to the pancake, due to the tube effect (Mezei *et al.*, 2014 ▸), in which the viewing angle is roughly parallel to the moderator walls, resulting in an enhanced depth of view. More details are given in the article by Zanini *et al.* (2018 ▸).

The flexibility of allowing efficient cold and thermal extraction for each beamport, which is such an attractive feature of the TDR moderators, is also achieved. Each beamport is aligned to point towards the nearest cold–thermal junction, indicated by a red spot in Fig. 13 ▸ for the instruments in the North sector. We recall that there are four of these focal points, defining the origin of the beamport axes in each of the four instrument sectors (North, West, South, East; see Fig. 1 ▸). The cold and thermal sources can be viewed almost perpendicularly for all beamports by tilting the guide axis by about 1° with respect to the beamport axis, which is compatible with the beamport insert geometry. An example of the view which can be achieved for the middle beamport of the North sector is illustrated with the light-blue and pink shaded areas in Fig. 13 ▸. The areas correspond to a view width of 7 cm, tilted by 1° with respect to the beamport axis around the beamport entrance window at 2 m from the focal point.

Finally, similar to the near-corner extraction of the pancake moderator, there is no clash of beamlines around the perpendicular to the proton beam, allowing the full complement of 42 beamports to be installed.

**Figure 14:**
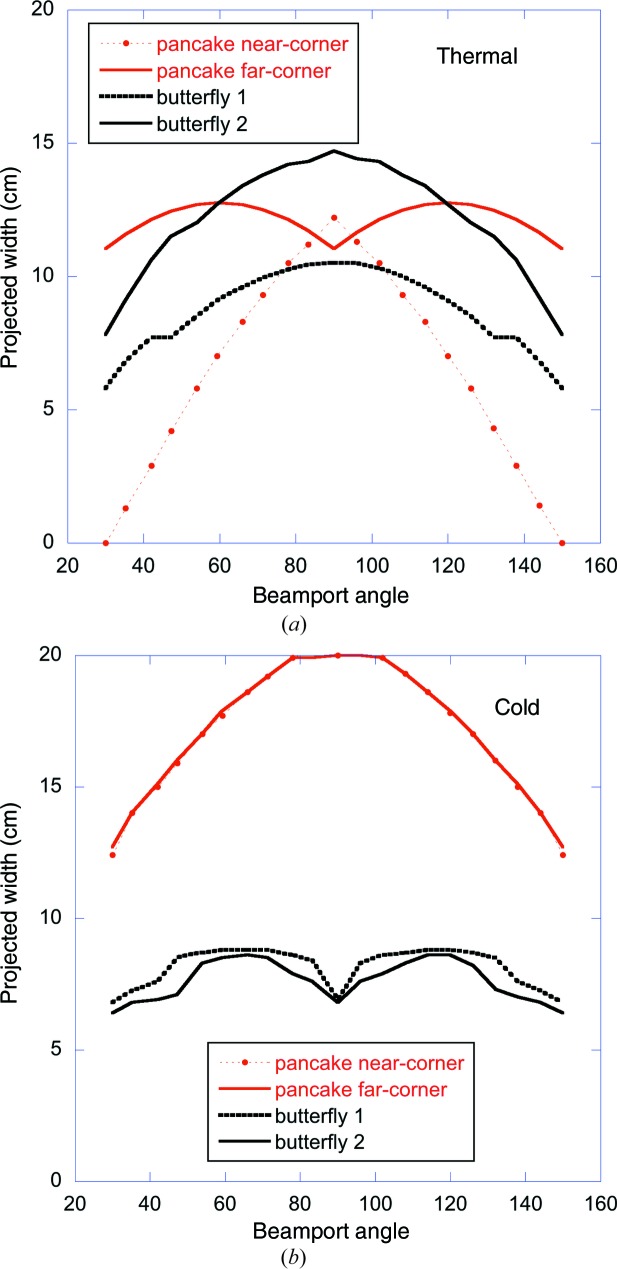
The projected width of the pancake and butterfly moderators as a function of beamport angle. (*a*) Thermal moderators. (*b*) Cold moderators. For the pancake thermal moderator, the width is measured from the focal point (as shown in Fig. 12 ▸) until the position where the moderator brightness has decreased by a factor of two, projected onto the viewed direction. All the other curves show the width simply defined by the geometry, starting from the focal point. The beamport angle is defined as counter-clockwise, with zero running along the transmitted proton beam. The widths on the other side of the proton beam are the same.

The projected widths of the cold and thermal parts of the pancake and butterfly moderator assemblies are shown in Fig. 14 ▸ as a function of beamport angle. As can be seen, the pancake geometry provides a very large viewable width of the cold moderator for all beamports. The projected width of the thermal moderator is seen to vary fairly weakly with angle for the far-corner extraction, but falls to zero in the case of near-corner extraction for beamports at the edge of the opening.

**Figure 15:**
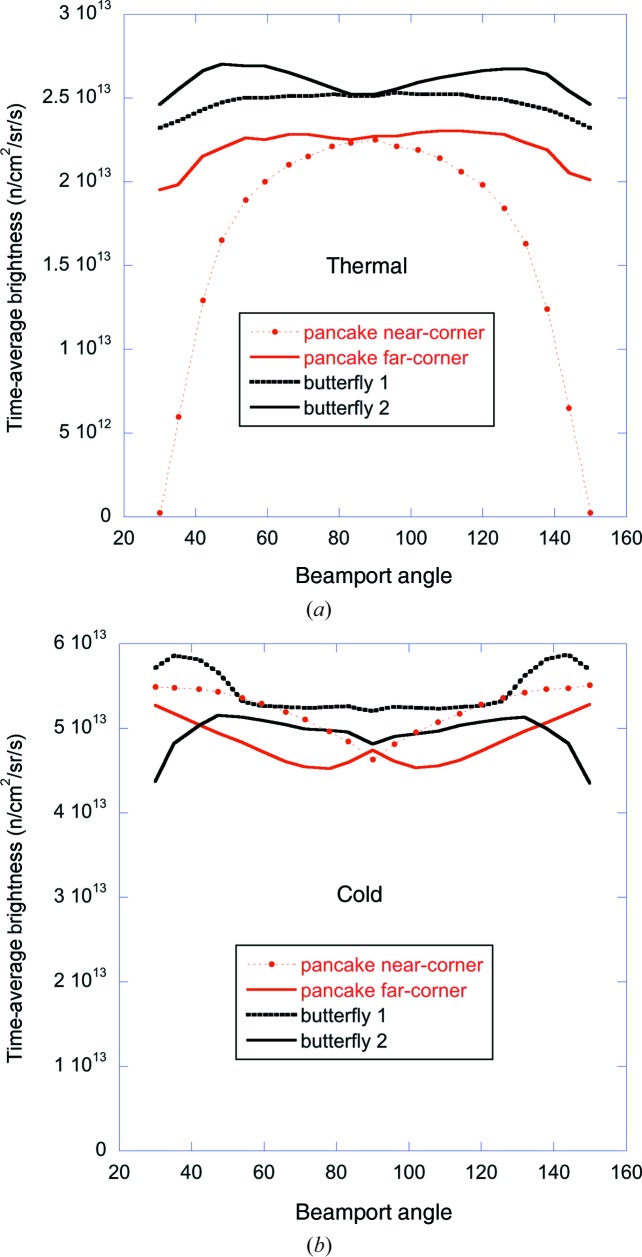
The time-averaged brightness of the pancake and butterfly moderators as a function of beamport angle, for a moderator height of 3 cm. (*a*) Thermal moderators. (*b*) Cold moderators. In all cases, the brightness is integrated over a projected area of 6 × 3 cm (H × V), starting within 1 cm horizontally of the focal point. The cold brightness is integrated over all energies up to 20 meV. The thermal brightness is integrated over energies from 20 to 100 meV. The beamport angle is defined as counter-clockwise, with zero running along the transmitted proton beam. The brightness values on the other side of the proton beam are the same.

The source brightness averaged over the most intense 6 cm width within the 7 cm wide bands indicated in Figs. 12 ▸ and 13 ▸ on the pancake, BF1 and BF2 moderators is shown in Fig. 15 ▸ as a function of beamport angle. The cold performance is seen to be similar for the four geometries, varying by about 10–20% as a function of angle and between the geometries.

The BF1 geometry gives the highest cold brightness, especially for beamlines around 45°. Note that for these beamlines there is an additional 10% gain in cold brightness to be had with the BF1 geometry, if the cold beam uses an area on the moderator face of only 3 cm width (Zanini *et al.*, 2018 ▸). Instruments using a reduced phase-space volume (such as single-crystal diffractometers or SANS instruments) are well placed to take advantage of this feature.

The thermal performance shows a strong preference for the butterfly geometry: the near-corner pancake geometry has catastrophically low brightness for the edge beamlines, while the far-corner pancake geometry has a more acceptable brightness, though still about 10–20% lower than the two butterfly geometries.

The BF1 and BF2 geometries thus provide good bispectral extraction for all beamports, with the BF1 geometry favouring the cold spectrum and the BF2 geometry favouring the thermal.

**Figure 16:**
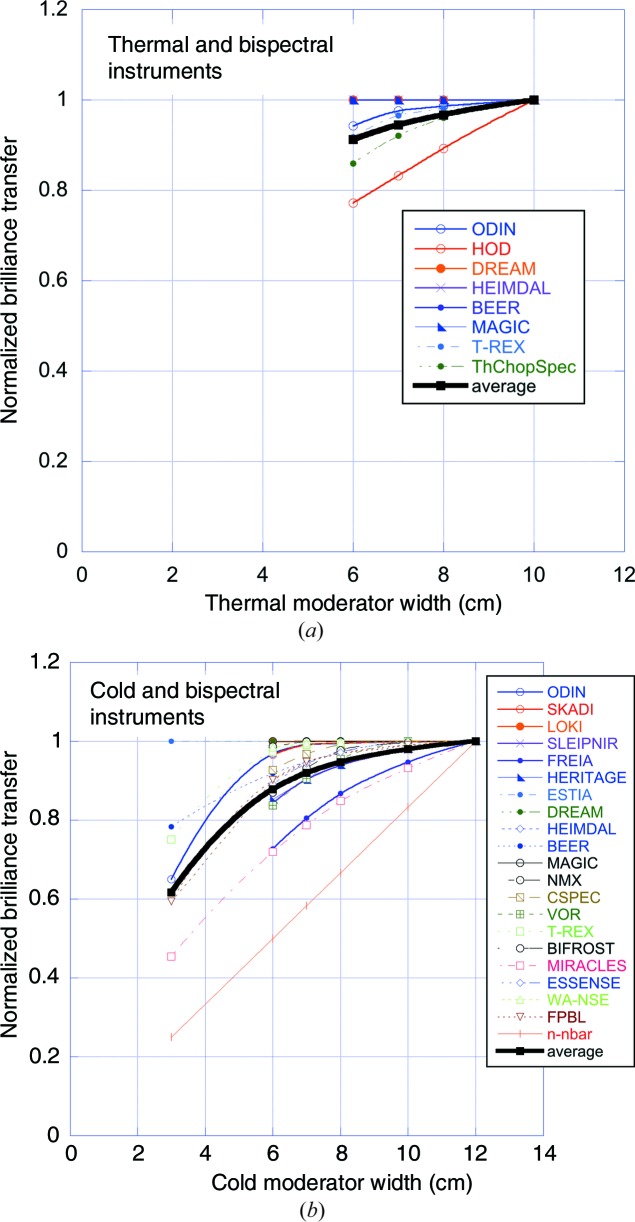
BT as a function of moderator width, (*a*) for instruments viewing the thermal moderator and (*b*) for instruments viewing the cold moderator. In both cases, bispectral instruments (*i.e.* instruments viewing both thermal and cold moderators) were given half weight when evaluating the average curves shown in the two plots.

As shown in Fig. 14 ▸, the projected moderator width of the various geometries considered varies strongly between them and also as a function of beamport angle. The reduction in moderator width associated with the move to the butterfly geometry reduces the horizontal illumination of the guides. A study was therefore performed to evaluate the impact on the BT, similar to the study outlined in the previous section on the vertical illumination. The impact on the BT of thermal moderator widths between 6 and 10 cm and cold moderator widths between 3 and 12 cm was evaluated. The results are summarized in Fig. 16 ▸.

The BT curves in Fig. 16 ▸ have been normalized to unity for a thermal moderator width of 10 cm and for a cold moderator width of 12 cm, as they are not expected to increase significantly for greater widths. These are the approximate widths which correspond to over-illumination of a 5–6 cm wide guide coated with an *m* = 2–3 supermirror starting at 2 m from the moderator. The useful moderator width is less for thermal neutrons, owing to the lower divergence which can be reflected by the supermirror coating.

When moving from the pancake to the butterfly geometries, the thermal moderator width of about 12 cm for the far-corner extraction remains roughly unchanged for BF2 and is reduced to about 9 cm for BF1. These widths in the two BF geometries remain sufficiently large that the guides are generally still in the over-illumination regime, as can be seen by the weak width dependence of the thermal BT in Fig. 16 ▸.

The cold moderator width is reduced rather more, from typically more than 15 cm to about 7–8 cm, and this does result in some under-illumination. The instrument-averaged cold BT curve in Fig. 16 ▸ indicates that the impact on the BT is of the order of 5–10%, which is small enough not to be a serious concern.

We emphasize that it is important to minimize this under-illumination by adapting the guide design to the dimensions of and distance to the source, as has been done for all the BT calculations shown here. If the guide geometry were left unchanged while reducing the moderator dimensions, the under-illumination problem would be significantly worse.

In terms of the intrinsic source brightness, the BF1 geometry slightly favours the cold over the thermal brightness, while providing similar projected widths of the two sources for all beamports. The BF2 geometry provides slightly higher thermal brightness at the expense of cold brightness, while providing a much larger projected width of the thermal moderator compared with the cold. The performances of the TDR, the 3 cm tall pancake, and the BF1 and BF2 moderators are summarized in Table 2 ▸. More detailed information on the BF moderator performance and geometry can be found in the article by Zanini *et al.* (2018 ▸).

## 8. Summary and conclusions   

Table 2 ▸ gives the viewed dimensions and brightness numbers of the cold and thermal moderators for the three moderator assemblies studied here, as well as the TDR configuration. The pancake moderator provides a large increase in both thermal and cold brightness compared with the TDR geometry. However, both beam extraction options for the pancake geometry have problems which compromise this performance gain: near-corner extraction results in unusably low thermal brightness for the edge beamlines, as reflected by its lower beamport-averaged thermal brightness, while far-corner extraction results in the loss of several beamlines around the perpendicular to the proton beam. The two butterfly geometries offer a solution to these difficulties, while further improving on the brightness gains compared with the TDR geometry. The BF1 variant favours the cold neutron performance, providing a 10% higher brightness than BF2 and a slightly larger projected width. The BF2 variant favours thermal neutron instruments, with a 5% brightness increase accompanied by an increased width.

The initial analysis of the butterfly geometry led to a decision to implement the BF2 variant of the butterfly, favouring thermal over cold performance. At the time of writing, the detailed engineering design of the moderators has been completed and fabrication has started. Subsequent further analysis has resulted in a decision to move to the BF1 geometry. This has a number of advantages: it reduces the lateral size of the full moderator assembly, matching it better to the fast-neutron production volume in the target. It increases the viewable width of the cold moderator, improving the horizontal illumination of the guides for the cold instruments. It also brings the central, most intense, part of the thermal moderator closer to the focal points, which were otherwise difficult to view in the BF2 geometry, as illustrated by the view windows indicated by the shaded areas in Fig. 13 ▸. As a result, when moving from BF2 to BF1 the brightness of the viewable area of the thermal moderator decreases by only 5%, while the cold moderator brightness increases by 10%, averaged over all beamports, as can be seen in Fig. 15 ▸ and Table 2 ▸. The change also results in roughly equal projected widths of the cold and thermal moderators, with a slightly increased cold width and a reduced thermal width. The increase in cold width provides a small additional gain, while the reduction in thermal width is not seen as problematic, as most thermal guides will still be over-illuminated in the horizontal direction.

The initial moderator assembly being built for day-one operation of the ESS has a BF2 geometry. It is planned, however, that the replacement moderator assembly will take the BF1 geometry. Owing to radiation damage, the moderator assembly will need to be replaced every year when operating at 5 MW accelerator power. The neutron instruments are all designing their beam extraction optics for the BF1 geometry.

Finally, the implementation of a single moderator assembly above the target which is able to serve all instruments in all sectors means that there is no need to install another moderator assembly below the target, as was planned for the TDR geometry. While the pancake and BF moderators were being evaluated, the installation of an identical, but taller, BF moderator below the moderator was considered in order to provide a lower-brightness option with better guide illumination, resulting in smoother divergence profiles or more phase space for instruments which need it. A height of 6 cm was foreseen for the lower moderator. A more complete analysis revealed that most instruments preferred the 3 cm moderator and the few which preferred the taller version only gained very slightly (<10%) in comparison. It was therefore decided not to install a lower moderator. This has several positive impacts: it reduces cost, it decreases the risk of moderator failure by reducing the overall complexity of the moderator systems and, importantly, it provides an additional upgrade path.

The upgrade path foreseen for most pulsed spallation sources is to build a second target station, so as to allow for the construction of new instruments with new capabilities. Such an upgrade has a similar cost to the initial target station construction budget and dilutes the power produced by the accelerator by needing to distribute it between two target stations. While such an upgrade is equally possible at the ESS, a more cost-effective path is also possible at the ESS by making use of the existing grid of 42 beamports, each of which can view either the top or bottom moderator. Allowing for a future, as yet unspecified, bottom moderator provides an elegant and flexible way of facilitating such an upgrade. By not installing instruments viewing the bottom moderator, no already installed instrument will need to be compromised in performance or moved to the other moderator to make way for the upgrade. It creates full freedom to design a moderator which can provide completely new capabilities for the ESS. Some ideas already exist for such upgrade paths, some of which can be combined:

(i) Large surface moderator. Possibly liquid D_2_, providing a very large area-integrated brightness of cold neutrons. Exploratory calculations were performed by Klinkby *et al.* (2014 ▸), indicating large gains in intensity.

(ii) High-brightness directional moderator. Neutronics calculations indicate that a rod-shaped *para*-H_2_ moderator can give a very large brightness increase in the direction of the rod axis (Zanini *et al.*, 2018 ▸). Such a moderator could serve a small number of beamports.

(iii) Very cold neutrons. An appropriately designed low-temperature volume moderator coupled to an effective reflector system could provide a large flux increase in neutron wavelengths in the 8–100 Å range. Potential applications include SANS, spin–echo, neutron microscopy and holography.

(iv) A beamline for neutron–antineutron (n-nbar) oscillations is currently being considered. This would be a single-purpose particle-physics experiment, which would benefit from a very large surface moderator with an intense spectrum of cold and very cold neutrons. The target monolith has been designed to allow one very large beam to be extracted towards an upgrade area allowing for a very long (200–300 m) beamline, as indicated in Fig. 1 ▸.

This list is by no means exhaustive but gives a flavour of the opportunities as they appear today. They may look very different in the future when an upgrade will need to be considered more carefully.

To summarize, we have demonstrated the gains in performance and flexibility which will be achieved by the use of flattened ‘butterfly’ moderators at the ESS. The design of the moderators has been an iterative process in which instrument performance, bispectral beam extraction and allowing a large number of instruments to view the moderators have all been driving factors.

The gains in source brightness by reducing the moderator height have been balanced against the resultant loss of brilliance transfer, by evaluating the performance of a full suite of instruments. As a result, a moderator height of 3 cm has been chosen, and the closest approach of the guide system to the moderators has been set to 2 m, representing the best compromise between instrument performance and the avoidance of unnecessary technical risk. Once the moderator height was chosen, work was concentrated on designing the best moderator shape for optimal beam extraction and flexibility, resulting in the choice of the butterfly configuration.

Another important result of this holistic approach to moderator and beam extraction design is the finding that a single moderator height, and hence a single moderator assembly above the target, is sufficient to provide optimal performance for all instruments. This opens up tremendous opportunities for future upgrades of the facility, by installing a qualitatively different moderator assembly below the target, without any changes needed to the target or beam extraction system.

The long-pulse nature of the ESS neutron source will allow an unprecedented level of flexibility for each instrument compared with other pulsed sources in adjusting the pulse width for all instruments to the needs of the experiment, without compromising on the peak brightness. The design of the moderator and beam extraction system further adds to this flexibility, by allowing each beamport to extract a cold, thermal or bispectral wavelength distribution, both for the day-one instrument suite and for the lifetime of the facility.

## Figures and Tables

**Table 1:** List of instruments used for the optimization study described here In most cases, the instrument description in the first column matches the instrument name in the TDR reference suite (Peggs, 2013 ▸). Instruments marked with an asterisk (*) in the first column are those which are substantially different from the TDR instruments. SLEIPNIR and HERITAGE are very similar to the TDR broad-band high-flux SANS and surface scattering instruments, respectively, in terms of phase-space requirements. HEIMDAL is similar to the TDR thermal powder diffractometer, with a SANS add-on to access multiple length scales. T-REX is similar to the TDR thermal chopper spectrometer, but employs a bispectral switch to extend its capabilities to the cold range. The n-nbar beamline has no equivalent in the TDR.

Instrument	*L*_m-s_	Beam area at sample (H × V)	Beam divergence at sample (H × V)	Wavelength range
Multi-purpose imaging ODIN	50 m	3 × 3 cm pinhole	At pinhole: ±0.7 × 0.7°	Bispectral, 1–7 Å
General-purpose polarized SANS SKADI	30 m	3 × 3 cm	±0.29 × 0.29°	Cold, 2–10 Å
Broad-band high-flux SANS LOKI	20 m	3 × 3 cm	±0.57 × 0.57°	Cold, 2–12 Å
Compact SANS SLEIPNIR*	16 m	2 × 2 cm	±0.86 × 0.86°	Cold, 3–19 Å
Horizontal reflectometer FREIA	27 m	0.4 × 4 cm	1.5 × 4°	Cold, 2–9.5 Å
Alternative horizontal reflectometer HERITAGE*	36 m	1 × 1 cm	±2 × 0.75°	Cold, 2–10 Å
Vertical reflectometer ESTIA	52 m	1 × 10 mm	±0.75 × 0.75°	Cold, 5–9 Å
Pulsed monochromatic powder diffractometer HOD	*L*_m-m_ 45 m	6 × 30 cm mono	At mono: ±0.5 × 0.5°	Thermal, 1.89 Å
Bispectral powder diffractometer DREAM	75 m	1 × 1 cm	±0.25 × 0.25°	Bispectral, 0.8–4.6 Å
Hybrid diffractometer HEIMDAL*	167 m	5 × 15 mm	±0.24 × 1.0°	0.6–2.3 Å
			±0.5 × 0.5°	4–10 Å
Materials science and engineering diffractometer BEER	157 m	5 × 10 mm	±0.14 × 0.86°	Bispectral, 0.5–3.8 Å
Single-crystal magnetism diffractometer MAGIC	150 m	1 × 1 cm	±0.2 × 0.2°	0.7–2.4 Å
			±0.5 × 0.5°	2.4–8 Å
Macromolecular diffractometer NMX	150 m	5 × 5 mm	±0.2 × 0.2°	Cold, 1.5–3.3 Å
Cold chopper spectrometer CSPEC	151 m	1.9 × 4 cm	±1 × 1°	Cold, 2–6 Å
Bispectral chopper spectrometer VOR	31 m	1 × 1 cm	±1 × 1°	Cold, 1–9 Å
Alternative bispectral chopper spectrometer T-REX*	150 m	1 × 3 cm	±1 × 1°	Bispectral, 0.8–7.2 Å
Thermal chopper spectrometer	160 m	3 × 3 cm	±1 × 1°	Thermal, 0.6–3 Å
Cold crystal-analyser spectrometer BIFROST	170 m	15 × 15 mm	±0.75 × 1°	Cold, 1.65–6.4 Å
Backscattering spectrometer MIRACLES	163 m	3 × 3 cm	±2.5 × 2.5°	Cold, 2–8 Å
High-resolution spin echo ESSENSE	27 m + 4 m	3 × 3 cm	±0.57 × 0.57°	Cold, 4–25 Å
Wide-angle spin echo WA-NSE	47 m + 4 m	1.5 × 6 cm	±0.5 × 1°	Cold, 2–10 Å
Fundamental and particle physics ANNI	30 m	6 × 6 cm	±0.57 × 0.57°	Cold, 3–8 Å
n-nbar beamline*	300 m	Moderator surface	Brightness × λ^2^	Cold, 0–20 Å

**Table 2:** Main parameters of the moderator geometries covered by this study

	TDR	Pancake near-corner	Pancake far-corner	Butterfly 1	Butterfly 2
Viewed height	12 cm	3 cm	3 cm	3 cm	3 cm
Horizontal opening	4 × 60°	2 × 120°	2 × 120°	2 × 120°	2 × 120°
Thermal brightness†	1.06 × 10^13^	1.64 × 10^13^	2.21 × 10^13^	2.47 × 10^13^	2.60 × 10^13^
Cold brightness‡	1.98 × 10^13^	5.23 × 10^13^	4.83 × 10^13^	5.45 × 10^13^	4.94 × 10^13^
Thermal width§	11 cm	6.4 cm	12.1 cm	8.7 cm	12.1 cm
Cold width§	12 cm	17.2 cm	17.2 cm	8.2 cm	7.6 cm

† The time-averaged spectral brightness, integrated over all energies from 20 to 100 meV, averaged over all beamports, the full moderator height and 6 cm width, chosen within the 7 cm ranges shown in Figs. 12 ▸ and 13 ▸ to maximize the brightness. In units of n cm^−2^ sr^−1^ s^−1^.

‡ As above, but integrated over all energies below 20 meV.

§ Projected onto the beamport normal and averaged over all beamports. The pancake thermal moderator widths correspond to the width needed to include the areas which are >50% of the most intense region, adjacent to the cold moderator, while the other widths correspond to the physical dimensions of the moderating volumes.

## Footnotes

2 Private communications in 2014 and 2015 from M. Strobl (ODIN), M. Morgano (ODIN), A. Jackson (LOKI, SKADI, SLEIPNIR), S. Jaksch (SKADI), H. Wacklin (FREIA), J. Stahn (ESTIA), S. Mattauch (HERITAGE), D. Lott (HERITAGE), A. Ioffe (HERITAGE), P. Henry (HOD), B. Rosendahl Hansen (HOD), C. Zendler (DREAM, MAGIC), W. Schweika (DREAM, MAGIC), X. Fabrèges (MAGIC), A. Goukassov (MAGIC), M. Christensen (HEIMDAL), E. Oksanen (NMX), P. P. Deen (VOR, thermal chopper spectrometer), N. Violini (T-REX), J. Voigt (T-REX), Th. Brückel (T-REX), W. Lohstroh (CSPEC), J. Okkels Birk (BIFROST), N. Tsapatsaris (MIRACLES), R. Lechner (MIRACLES), H. N. Bordallo (MIRACLES), S. Pasini (ESSENSE, WA-NSE), M. Monkenbusch (ESSENSE, WA-NSE), C. Theroine (ANNI) and G. Greene (n-nbar).

## References

[bb1] Baldo-Ceolin, M. *et al.* (1994). *Z. Phys. C*, **63**, 409–416.

[bb2] Batkov, K., Takibayev, A., Zanini, L. & Mezei, F. (2013). *Nucl. Instrum. Methods Phys. Res. A*, **729**, 500–505.

[bb3] Bentley, P. M. & Andersen, K. H. (2009). *Nucl. Instrum. Methods Phys. Res. A*, **602**, 564–573.

[bb4] Bentley, P. M. & Klinkby, E. (2015). *Proposed Baseline Change of Neutron Beam Origin Points.* ESS Internal Report. ESS, Lund, Sweden.

[bb5] Bertelsen, M. (2017). *Nucl. Instrum. Methods Phys. Res. A*, **867**, 195–203.

[bb6] Bertelsen, M. & Lefmann, K. (2016). *Nucl. Instrum. Methods Phys. Res. A*, **830**, 313–324.

[bb7] Carpenter, J. M. & Mildner, D. F. R. (1982). *Nucl. Instrum. Methods Phys. Res.* **196**, 341–348.

[bb8] Grammer, K. B. *et al.* (2015). *Phys. Rev. B*, **91**, 180301.

[bb9] Institut Laue–Langevin (2008). *The ILL Yellow Book.* ILL, Grenoble, France.

[bb10] Kai, T., Harada, M., Teshigawara, M., Watanabe, N. & Ikeda, Y. (2004). *Nucl. Instrum. Methods Phys. Res. A*, **523**, 398–414.

[bb11] Klinkby, E., Batkov, K., Mezei, F., Schönfeldt, T., Takibayev, A. & Zanini, L. (2014). *arXiv*: 1401.6003v1.

[bb12] Lefmann, K. & Nielsen, K. (1999). *Neutron News*, **10**(3), 20–23.

[bb13] Liouville, J. (1838). *J. Math. Pure Appl. Ser. 1*, **3**, 342–349.

[bb14] Magán, M., Sordo, F., Zanini, L., Terrón, S., Ghiglino, A., Martínez, F., deVicente, J. P., Vivanco, R., Perlado, J. M., Bermejo, F. J., Mezei, F. & Muhrer, G. (2013). *Nucl. Instrum. Methods Phys. Res. A*, **729**, 417425.

[bb17] Mezei, F. & Russina, M. (2003). US Patent No. US 20050157831 A1.

[bb15] Mezei, F., Zanini, L., Takibayev, A., Batkov, K., Klinkby, E., Pitcher, E. & Schönfeldt, T. (2014). *J. Neutron Res.* **17**, 101–105.

[bb16] Milstead, D. (2015). *arXiv*: 1510.01569v1 [physics.ins-det].

[bb18] Peggs, S. (2013). *ESS Technical Design Report.* ESS, Lund, Sweden.

[bb20] Šaroun, J. & Kulda, J. (2008). *Restrax* *Modern Developments in X-ray and Neutron Optics*, edited by A. Erko, M. Idir, T. Krist & A. Michette, pp. 57–68. Berlin: Springer.

[bb21] Schönfeldt, T. (2016). *Advanced Neutron Moderators for the ESS*. Technical University of Denmark (DTU), Kongens Lyngby, Denmark.

[bb22] Wechsler, D., Zsigmond, G., Streffer, F. & Mezei, F. (2000). *Neutron News*, **11**(4), 25–28.

[bb23] Willendrup, P., Farhi, E., Knudsen, E. B., Filges, U. & Lefmann, K. (2014). *J. Neutron Res.* **17**, 35–43.

[bb25] Zanini, L., Andersen, K. H., Batkov, K., Klinkby, E. B., Mezei, F., Schönfeldt, T. & Takibayev, A. (2018). In preparation.

[bb27] Zanini, L., Batkov, K., Klinkby, E. B., Mezei, F., Pitcher, E., Schönfeldt, T. & Takibayev, A. (2014). Proceedings of ICANS-XXI, 29 September–3 October 2014, Mito, Japan, pp. 126–133.

[bb28] Zanini, L., Batkov, K., Klinkby, E. B., Mezei, F., Schönfeldt, T. & Takibayev, A. (2016). Book of Abstracts of ICANS-XXII, 27–31 March 2017, Oxford, UK, p. 39.

[bb29] Zanini, L., Batkov, K., Mezei, F., Takibayev, A., Klinkby, E. B. & Schönfeldt, T. (2015). Proceedings of the AccApp’15 Conference, 10–13 November 2015, Washington DC, USA, pp. 272–277.

